# Sustainable Durian Rind Carboxymethyl Cellulose/Poly(vinyl) Alcohol Hydrogels Synthesis for Enhancing Crosslinking and Release Kinetics Efficiency

**DOI:** 10.3390/gels11090728

**Published:** 2025-09-11

**Authors:** Kanticha Pratinthong, Rangsan Panyathip, Sarinthip Thanakkasaranee, Kittisak Jantanasakulwong, Wirongrong Tongdeesoontorn, Duangjai Noiwan, Thomas Karbowiak, Chitsiri Rachtanapun, Pornchai Rachtanapun

**Affiliations:** 1 Division of Packaging Technology, School of Agro-Industry, Faculty of Agro-Industry, Chiang Mai University, Mae Hia, Chiang Mai 50100, Thailand; kanticha25.p@gmail.com (K.P.); sarinthip.t@cmu.ac.th (S.T.);; 2 Division of Physics, Faculty of Science and Technology, Rajamangala University of Technology Thanyaburi, Pathum Thani 12110, Thailand; rangsanpanyatip@gmail.com; 3 Center of Excellence in Agro Bio-Circular-Green Industry (Agro BCG), Chiang Mai University, Chiang Mai 50200, Thailand; 4 Center of Excellence in Materials Science and Technology, Faculty of Science, Chiang Mai University, Chiang Mai 50200, Thailand; 5 School of Agro-Industry, Mae Fah Luang University, 333 Moo 1 Tasud, Chiang Rai 57100, Thailand; wirongrong.ton@mfu.ac.th; 6 Research Group of Innovative Food Packaging and Biomaterials Unit, Mae Fah Luang University, 333 Moo 1 Tasud, Chiang Rai 57100, Thailand; 7 Department of Postharvest Technology, Faculty of Engineering and Agro-Industry, Maejo University, Chiang Mai 50290, Thailand; duangjai_nw@mju.ac.th; 8 Institut Agro, Université Bourgogne Europe, INRAE, UMR PAM, F-21000 Dijon, France; thomas.karbowiak@institut-agro.fr; 9 Department of Food Science and Technology, Faculty of Agro-Industry, Kasetsart University, Bangkok 10900, Thailand; chitsiri.t@ku.th; 10 Center for Advanced Studies for Agriculture and Food, Kasetsart University, Bangkok 10900, Thailand

**Keywords:** agricultural waste, biomaterials, crosslink, hydrogel films, methylene blue

## Abstract

This study developed hydrogels from durian rind-derived carboxymethyl cellulose (CMC_d_) blended with poly(vinyl) alcohol (PVA) for biomedical applications. The influence of NaOH concentration (10–60% *w*/*v*) on the degree of substitution (DS) of CMC_d_ and the crosslinking properties of the resulting hydrogels was examined. Durian rind, a biodegradable and renewable resource, was transformed into CMC_d_ with DS values ranging from 0.17 to 0.94. The highest yield (230.96%) was achieved using 30% NaOH (CMC_d_-30). This CMC_d_-30 was combined with PVA and crosslinked using citric acid to form a hydrogel with maximum crosslinking efficiency (86.16%). The resulting CMC_d_-30/PVA hydrogel exhibited a high swelling ratio (125.54%), reflecting its superior water absorption and functional group availability—key traits for biomedical use. Methylene blue (MB) release from the hydrogel extended up to 1440 min, confirming its drug delivery potential. Overall, the CMC_d_-30/PVA hydrogel demonstrated promising biocompatibility potential and performance, making it a promising candidate for wound dressings and controlled drug delivery systems. This work highlights the potential of agricultural waste valorization in developing sustainable and efficient biomaterials for pharmaceutical and medical applications.

## 1. Introduction

Agricultural waste is an abundant source of polysaccharides, composed of carbohydrate polymers for more than 90% (*w*/*w*) and functionalized as cellulose in biomaterials. This is particularly useful in addressing environmental concerns through the transformation of plant waste into value-added products. Durian is widely well known as the “king of fruits” from Thailand with its distinctive odor, round shape, and unique taste. Durian fruit is usually enclosed in a strong, thorn-covered rind, which may be used as a renewable and biodegradable source of cellulose and polysaccharides [1,2].

Durian peel accounts for nearly half of the fruit’s mass and represents a significant stream of agro-waste in Southeast Asia, generating over 400 kt yr^−1^. Its cellulose fraction (30–35 wt%), low lignin (5–10 wt%), and minimal inorganic residue (<3 wt%) make it an ideal candidate for carboxymethylation under mild conditions. The porous architecture of the peel enhances chemical accessibility, resulting in CMC products with high degrees of substitution and viscosities comparable to those derived from commercial wood pulp or sugarcane bagasse. This valorization approach not only reduces environmental burden but also delivers functional biopolymers at lower cost and with a smaller processing footprint. Durian peel feedstock, an abundant source of agro-waste in Southeast Asia, can be procured at roughly USD 5–10 per ton, which is vastly lower than bleached wood pulp (USD 500–700 per ton) or sugarcane bagasse (USD 20–40 per ton), resulting in estimated CMC production costs of ~USD 1.8 per kg versus USD 3.2 per kg for wood pulp CMC and USD 2.4 per kg for bagasse CMC. Despite its low cost, CMC_d_-30 achieves a degree of substitution of 0.94 and a solution viscosity of 1560 mPa·s, matching or exceeding wood pulp CMC (DS = 0.70; 1400 mPa·s) and bagasse CMC (DS = 0.80; 1500 mPa·s. In contrast, bamboo-derived CMC typically exhibits lower DS (0.60–0.75) and viscosities (1200–1300 mPa·s) due to higher lignin content and denser fiber structure [1]. These comparisons underscore the notion that durian peel not only reduces raw-material costs by an order of magnitude but also delivers CMC with superior functional properties.

Previously, CMC products were studied and prepared from several cellulose sources with various raw materials, such as sugar beet pulp [3], sago palm [4], *Mimosa pigra* peel [5], papaya peel [6,7], rice stubble [8], *Asparagus* stalk end [9], bagasse and palm bunch pulp [10], Cavendish banana pseudo stem [11], durian rind [1,12], corncob [13], Nata de Coco [14,15], young Palmyra palm fruit [16], and young and mature coconut coir [17]. The cellulose of durian rinds is converted into carboxymethyl cellulose (CMC) through an etherification process under alkaline conditions, enhancing its properties for functionalization [2,5].

CMC is prepared by the activation of cellulose with NaOH in aqueous media, reacting with monochloroacetic acid as the etherifying agent [18]. CMC is a cellulose derivative derived from natural fibers. It displays several beneficial properties, including high water solubility, thermal stability, and biocompatibility [19]. It is used as a stabilizer, viscosity modifier, food-thickening agent, and biomaterial in agricultural and medical applications [20,21]. CMC reveals high solubility in water, coupled with increased viscosity. It is used as a stabilizing agent in the food industry, highlighted in ice cream, and as a coating for drug capsules [22]. With its unique CMC properties, it is widely utilized in several industries, including laundry, paint, glue, textiles, paper, ceramics, and food, as well as medicine applications [21,23]. Extracted durian rind cellulose can be converted into durian rind carboxymethyl cellulose (CMC_d_) via carboxymethylation, yielding a material with superior solubility, processability, and functional versatility [1]. The cellulose extracted from durian rind is ideal to produce CMC_d_ material, used in several industries [24]. The cellulose extracted from durian rind serves as a sustainable source for the production of CMC_d_, which can be used in various industrial and pharmaceutical applications due to its biocompatibility, non-toxicity, and hydrogel capacity, making it promising for biomedical applications [25]_._ The development of hydrogels from CMC_d_ offered a sustainable and cost-effective approach for advanced material synthesis, addressing the agricultural waste issue management [26]. Hydrogel materials have received considerable attention due to their compatibility, biological stability, favorable mechanical properties, and promising physical attributes, as well as ease of processing and production [27,28]. Semi-synthetic water-soluble cellulose polymers, CMC, are widely employed to form hydrogels for application in medicine and pharmaceuticals [29]. The CMC_d_ production from agricultural waste materials like durian peels is an avenue for further hydrogel development [30]. Using biodegradable cross-linking agents, CMC_d_ could be harnessed to produce biologically compatible medical materials, offering promising prospects for future research and application.

In the previous study, cellulose nanofibers (CNF) derived from durian peels were adapted to enhance the mechanical strength and porosity of aerogels, offering an eco-friendly solution for the development of high-performance materials [31]. In the pharmaceutical field, durian-derived cellulose was explored by applying it in 3D printing, producing pectin/cellulose hydrogels as bioinks. These hydrogels enable the creation of smooth, uniform printed structures, making them suitable for biomedical and pharmaceutical applications [32]. CMC_d_ from durian peels was employed in the personalization of theophylline films, showing the compatibility for customized 3D-printed pharmaceutical products [33]. Corresponding to the promising properties of hydrogel, CMC was incorporated with poly(vinyl) alcohol (PVA) to form the CMC/PVA hydrogel films, applied in order for the adsorbent materials to remove the methylene blue (MB) from aqueous solutions. The results were indicated by the ion-exchanging mechanisms used for the MB removal in these hydrogel films, highlighting the potential of CMC/PVA films as reusable adsorbent material for wastewater treatment applications [34]. Additionally, the CMC/PVA hydrogel films were studied by a releasing mechanism of triamcinolone acetonide (TAA) drug, enhancing anti-inflammatory effectiveness for wound healing within 24 h of medical treatment. The CMC/PVA hydrogel films increased TAA absorption with higher crosslinker density, gradually releasing TAA and increasing the efficiency of anti-inflammatory activity [25].

Contrary to previous efforts that exploited durian-derived cellulose nanofibers to reinforce aerogels, pectin/cellulose bioinks for 3D printing, personalized theophylline films, and standard CMC/PVA blends for pollutant removal and drug delivery, this study introduces the systematic modulation of DS in CMC from durian peel via NaOH concentrations (10–60% *w*/*v*). This approach allows for the precise tuning of hydrogel network swelling and hydrophilicity, which (i) accelerates methylene blue chemisorption kinetics and enhances adsorption capacity, and (ii) enables customizable drug-release profiles. By unifying high-performance pollutant uptake and adjustable therapeutic delivery in a single, fully biodegradable CMC_d_/PVA film, this study addresses the gap in dual-function sustainable materials and paves the way for multifunctional biomedical and environmental applications.

In this study, we aimed to develop the properties of CMC_d_/PVA hydrogel films fabricated using CMC_d_ from biodegradable materials incorporated with PVA. The CMC_d_ structure was modified by the degree of substitution (DS) with NaOH (10–60% *w*/*v*), introducing the swelling properties enhancement of hydrogel films. These swelling properties contributed to their capability as an efficient alternative absorbent material, as observed through the release kinetics model of MB adsorption capacities in the chemisorption process of the provided hydrogel, confirming their potential suitability for drug-releasing applications [35].

## 2. Results and Discussion

### 2.1. Durian Rind Cellulose and CMC_d_ Characterization

#### 2.1.1. Degree of Substitution and Percent Yield of CMC_d_

**Figure 1:**
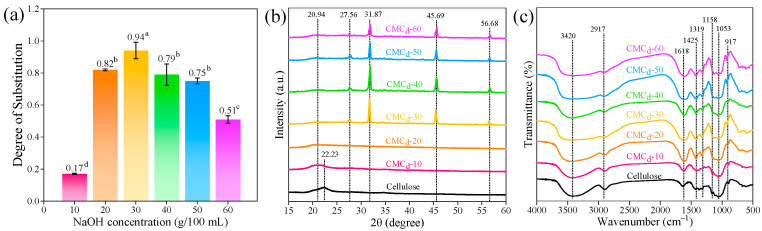
(**a**) DS of CMC_d_, (**b**) XRD spectra, and (**c**) FTIR chemical functional groups of the extracted durian rind cellulose and CMC_d_. Note: values indicated with the same letters are not significantly different at *p* ≤ 0.05.

Figure 1a shows the correlation between the NaOH concentrations and the DS of CMC_d_ produced from durian rind [1,21]. Using the NaOH concentration at 10% *w*/*v* induced the DS at 0.17, then reached 0.82 with the increased NaOH concentration at 20% *w*/*v*. The highest DS (0.94) was indicated at 30% *w*/*v* of NaOH concentration addition, while adding more NaOH concentration at 40–60% *w*/*v*, DS declined from 0.79 to 0.51. Corresponding to the DS results of CMC_d_, the optimal NaOH concentration for achieving CMC_d_ with the highest DS was represented at 30% *w*/*v*. Using a NaOH concentration of more than 30% *w*/*v* resulted in a reduction in DS efficacy [14,36]. As NaOH concentration increases beyond 30% *w*/*v*, DS drops from 0.94 to 0.51. This decline arises because excessive alkali leads to NaHCO_3_ formation, which (i) consumes free OH^−^, (ii) buffers the medium at sub-optimal pH for etherification, and (iii) diverts monochloroacetic acid toward side-product formation and precipitation. Consequently, fewer reactive sites on the cellulose backbone undergo substitution, and the overall DS decreases. In this part, the low NaOH concentrations resulted in a lower DS due to insufficient hydroxide ions available to promote effective etherification of cellulose. Conversely, adding NaOH concentration at 30% *w*/*v* facilitated the highest DS at 0.94, enabling an optimal substitution condition of hydroxyl groups with carboxymethyl groups, while the increasing NaOH concentration at 40–60% *w*/*v* led to a decline in DS. These results were caused by the degradation of cellulose and the formation of NaHCO_3_ during the substitution process.

**Scheme 1:**
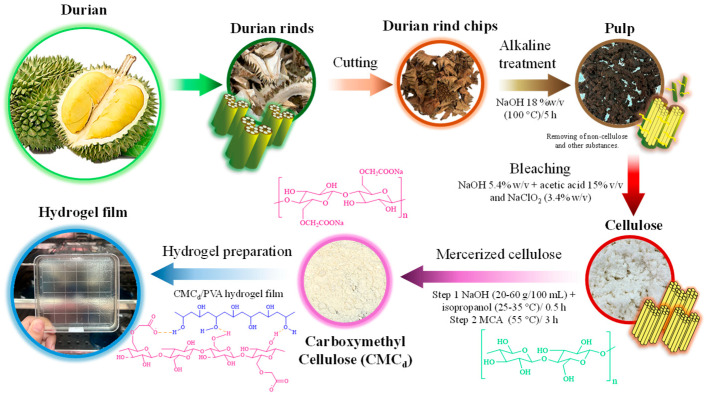
Preparation of hydrogel using CMC_d_ from durian rind cellulose combined with PVA.

By the CMC_d_ preparation in Scheme 1, durian rind cellulose was transformed into the structure of CMC_d_. The CMC_d_ yield was calculated using the weight ratio between the final product and initial raw material, showing the enhancement of yield to 89.75, 144.87, 217.04, 220.69, 230.96, and 232.52% for CMC_d_-10, CMC_d_-20, CMC_d_-30, CMC_d_-50, and CMC_d_-60, respectively.

The varied CMC_d_ yields were influenced by the DS results alongside the increment of NaOH concentration, contributing via the efficiency of the etherification process. At low NaOH concentrations of 10–20% *w*/*v*, the reaction introduced the low concentration of hydroxide ions with cellulose, resulting in low DS values of 0.17–0.82 with yields of 89.75 to 144.87%. When synthesizing CMC at 10% (*w*/*v*) NaOH, the limited availability of OH^−^ results in partial cellulose activation, yielding a DS of 0.25. Consequently, a significant fraction of cellulose remained low-substituted, which, together with CMC losses during washing and filtration, contributed to a lower overall recovery of 89%. Earlier reports observed yields below 90% at NaOH < 12% due to incomplete etherification and wash losses, while the deficit was attributed to the poor solubility of low-DS fractions and side-product formation under undersaturated alkali conditions.

With the 30% *w*/*v* NaOH concentration, the durian cellulose fibers were optimally swollen and activated, promoting the highest substitution of hydroxyl groups with carboxymethyl groups, reflecting the maximum DS at 0.94, and the increased CMC_d_ yield reached 217.04%. The CMC_d_ yield rose marginally at NaOH levels of 50–60% (*w*/*v*). Beyond this optimal range, however, additional NaOH led to over-substitution and the precipitation of residual salts, causing the overall yield to plateau. Thus, the appropriate concentrations of NaOH induce the cellulose breakdown and increase the yield via suitable DS. However, over-alkalinization hinders the chemical modification, affecting the decoupled yield from actual substitution efficiency.

#### 2.1.2. XRD Analysis of CMC_d_

According to the DS analysis in the previous section, the structures of durian rind cellulose and CMC_d_ obtained at various NaOH concentrations were analyzed using XRD [37]. Figure 1b shows the XRD results of extracted cellulose from durian rinds, revealing the crystalline structure of the cellulose after treatment with NaOH. XRD patterns of natural cellulose exhibit two sharp diffraction peaks at 2θ = 22.23°, corresponding to the (2 0 0) crystallographic planes of cellulose II [38], indicating the coexistence of crystalline and amorphous domains in the sample [39]. This high-intensity peak is assigned to (2 0 0), indicating an arrangement of the cellulose fibers. The existence of this peak confirmed the inherent nature structure of untreated cellulose [40]. CMC_d_ was obtained through the carboxymethylation reaction in an alkaline medium. The NaOH concentration significantly influenced the crystalline structure of CMC_d_. The crystalline arrangement of CMC_d_ was disrupted by adding more NaOH concentration. XRD patterns of CMC_d_ showed broadened and decreased intense peaks in CMC_d_-10 and CMC_d_-20 conditions, indicating the crystallinity reduction. This result suggested the CMC_d_ cellulose II showed a more amorphous structure formation. The broader peaks (Figure 1b) reflected the disordered arrangement of the internal structure, caused by chemical alterations in the cellulose structure during carboxymethylation [41]. The XRD results demonstrated that NaOH played a crucial role in the transformation of cellulose crystal structure during the CMC_d_ synthesis. At higher NaOH concentrations (30–60% *w*/*v*), the CMC_d_ samples exhibited a significant reduction in crystallinity. The XRD patterns showed weakened cellulose I (110) and (200) peaks at 2θ ≈ 20.24° and 22.23°, respectively, and the emergence of a broad amorphous halo around 18°, consistent with lattice disruption due to extensive carboxymethylation (DS = 0.51–0.94). The XRD peaks at 27.56°, 31.87°, 45.69°, and 56.68° correspond to sodium chloride (NaCl), a byproduct of the reaction between NaOH and MAC during the carboxymethylation process. These XRD peaks matched with JCPDS Card No. 05-0628 for NaCl, ascribing to (1 1 1), (2 0 0), (2 2 0), and (2 2 2), respectively [42,43]. The increasing intensity of these peaks with rising degree of substitution (DS = 0.51–0.94) confirms that more residual NaOH leads to greater NaCl formation within the CMC_d_ samples.

#### 2.1.3. Analysis of the Chemical Functional Group of CMC_d_

During the synthesis of CMC_d_, NaOH first deprotonates the cellulose hydroxyl groups to form alkoxide intermediates (–O^−^ Na^+^), which then undergo nucleophilic substitution with MCA, introducing carboxymethyl (–CH_2_COO^−^) groups into the cellulose backbone. In this process, NaOH serves solely as the alkaline medium, whereas the substitution itself occurs with monochloroacetate [44]. With the influence of NaOH concentration on the degree of substitution (DS) within the functional groups of CMC_d_, Figure 1c displays the chemical structure of durian rind cellulose and CMC_d_, analyzed by the vibrational transition from molecules using FTIR [45].

The primary chemical modification in the CMC_d_ synthesis involved the partial substitution of hydroxyl groups (–OH) in the cellulose structure with carboxymethyl groups (–CH_2_COOH) [46]. This modification significantly influenced the properties and chemical structure of cellulose. Durian rind cellulose structure with linear polysaccharide is composed of β–D–glucose units, linked by glycosidic bonds at carbon positions 1 and 4 as a stable crystalline structure via strong hydrogen bonding between the molecular chains [47]. Durian rind cellulose is insoluble in water and chemically inert; however, its functional groups were modified through varying NaOH concentrations (CMC_d_-10 to CMC_d_-60), resulting in the transformation of the cellulose structure into CMC_d_ [48].

The DS resulting from the NaOH-mediated substitution of hydroxyl groups in the cellulose structure influences the chemical functional groups in CMC_d_. This modification introduces carboxymethyl (–CH_2_COOH) groups into the cellulose backbone, as shown in Figure 1c, and is analyzed by FTIR. Durian rind cellulose possesses a polycrystalline structure with insolubility in water and chemical stability, achieved by the strong hydrogen bonds from its molecular chains [49]. CMC_d_ consists of carboxymethyl groups, which are introduced by modifying the hydroxyl groups of cellulose. These substitutions make CMC_d_ more amorphous and water-soluble, which differentiates it from durian rind cellulose. The FTIR results revealed the chemical functional group transformation between durian rind cellulose and CMC_d_ from the DS process [50]. The carboxymethyl groups of CMC_d_ were applied in various industrial applications, such as thickeners, gelling agents, and water retention agents [20,21]. These FTIR results of CMC_d_ were reflected in the successful modification of carboxymethyl groups in CMC_d_ in structure [51]. The functional groups of CMC_d_ from FTIR spectra were identified at 3420 and 2917 cm^−1^, assigned to the stretching vibration of O–H and C–H [25], respectively. The vibrations at 1618, 1425, 1319, and 1158 are identified for O–H adsorbed water, CH_2_ in-plane vibrational, CH_2_ wagging, and asymmetric stretching of a C–O–C pyranose ring [25,52,53,54,55,56], respectively. The vibrational modes at 1053 and 917 cm^−1^ are classified as the molecule stretching of –C–O (C3–OH secondary alcohol) and –C–O stretch (C6–OH primary alcohol), respectively [55]. The consequence of the FT-IR analysis demonstrated that hydroxyl groups in the cellulose were successfully replaced with carboxymethyl groups, confirming the occurrence of etherification related to the DS factor. The alkaline activation induced by NaOH revealed the chemical transformation of cellulose into Cellulose II, as indicated by the reduction in crystallinity during the carboxymethylation of CMC_d_.

#### 2.1.4. Morphology of CMC_d_

**Figure 2:**
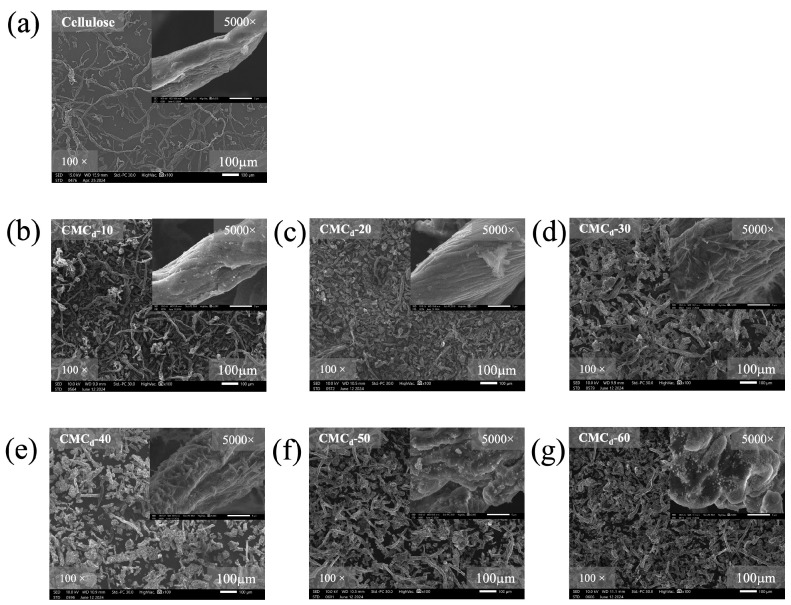
Morphological results of (**a**) durian rind cellulose fibers and CMC_d_ (**b**–**g**) prepared from durian rind cellulose with various NaOH concentrations for 10–60% *w*/*v* (recorded at 5000× magnification).

The SEM results showed the morphological analysis of cellulose fibers in CMC_d_ morphology (Figure 2), revealing the notable changes in fiber surface structure and arrangement of fiber organization. In Figure 2a, the SEM image of durian rind cellulose fibers shows those that exhibit elongated, irregularly arranged structures with a relatively rough surface texture, which represents the natural cellulose morphology before the chemical modification process. The morphology of CMC_d_ fibers was treated with NaOH concentrations from 10 to 60% *w*/*v*. By the NaOH concentrations at 10–20% *w*/*v* of Figure 2b,c, the surface structure of CMC_d_ was similar to the natural cellulose morphology [57]. As the NaOH concentration increased from 30% to 60% *w*/*v* (Figure 2d–g), significant morphological changes occurred, resulting in randomly oriented cellulose fibers with altered surface structure. These CMC_d_ resulted in obviously modified cellulose fibers, emphasizing the improvement of CMC_d_ quality by optimizing the morphological properties [58].

The SEM results indicated that durian rind cellulose was transformed into CMC_d_ samples, showed notable alterations, and was randomly dispersed in external morphology [59,60]. The fibers showed surface damage and irregularities of fibers, indicating the potential for more swelling and the degradation process of fibers [61]. These alterations reflected the chemical modifications on the fiber structure, separating the residual cellulose fibers from CMC_d_ product [62].

In addition, the SEM results of CMC_d_ with higher magnification indicated the characteristics of fibers in detail, with the fiber structure about 13 µm in size diameter [63]. The natural cellulose fibers are depicted as long, complex, and irregularly arranged fibers with a rough surface, as shown in Figure 2b–g [64]. Hence, the CMC_d_ derived from durian rinds provided valuable insights into the morphological changes in cellulose fibers, emphasizing how the modification process affected the structural integrity of the fibers. Moreover, the SEM results revealed significant morphological alterations in the cellulose fibers following NaOH treatment and subsequent carboxymethylation, such as surface damage and irregular structures. These observations are consistent with FT-IR and XRD results, both of which support the disruption of hydrogen bonding and crystalline structure, confirming successful modification of the cellulose into CMC_d_.

#### 2.1.5. Viscosity and Water Solubility of CMC_d_

**Figure 3:**
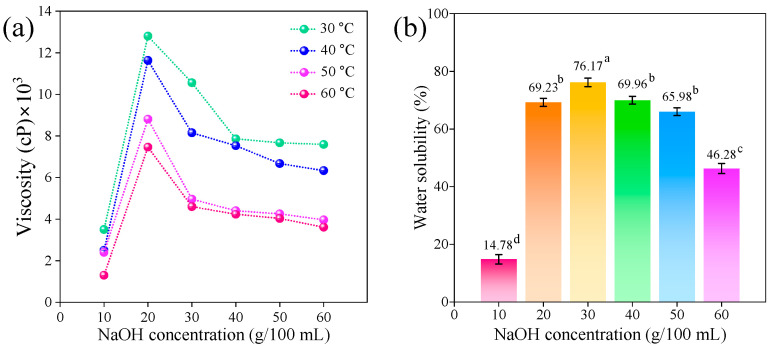
(**a**) Viscosity behavior and (**b**) water solubility of CMC_d_ at varying NaOH concentrations. Note: values indicated with the same letters are not significantly different at *p* ≤ 0.05.

Figure 3a shows the viscosity behavior of CMC_d_ samples at varying NaOH concentrations (10–60% *w*/*v*) and temperatures ranging from 30 to 60 °C. The viscosity was reduced by increasing the NaOH concentrations to higher than 20% *w*/*v* [65]. The increment of temperatures led to a reduction in the overall CMC_d_ viscosity. The viscosity testing of CMC_d_ exhibited the highest viscosity for all NaOH concentrations, while the overall viscosity was significantly reduced at 60 °C [17,35]. This factor contributed to the disruption of molecular interactions within the polymer structure in the CMC_d_ at higher NaOH concentrations and temperatures [66].

In Figure 3b, the water solubility of CMC_d_ increases from 14.78% to 76.17% as a result of enhanced hydrophilicity, conferred by its cellulose backbone bearing abundant carboxymethyl and residual hydroxyl groups. The high cellulose content provides multiple hydrogen-bonding sites with water, while the elevated degree of substitution reduces crystallinity and increases amorphous regions, collectively facilitating water penetration and dissolution [67,68]. The CMC_d_-30 was suggested as the optimal NaOH concentration with the highest solubility of CMC_d_ at 76.17%, obtained by the most favorable balance between solubility and structural integrity. The higher NaOH concentrations at 40 to 60% *w*/*v* exhibited continuously diminished solubility because the dissolution of the polymer in water was impeded by the crystallinity of CMC_d_. The synthesized CMC_d_ prepared with 30% NaOH concentration exhibited the highest viscosity and water solubility, indicating an optimal balance between structural integrity and hydrophilicity. These properties are consistent with the enhanced degree of substitution (DS) achieved under alkaline activation, as supported by XRD patterns and FTIR spectra that demonstrated reduced crystallinity and the formation of a more amorphous structure.

#### 2.1.6. Color Measurement of Cellulose Fibers and CMC_d_

**Figure 4:**
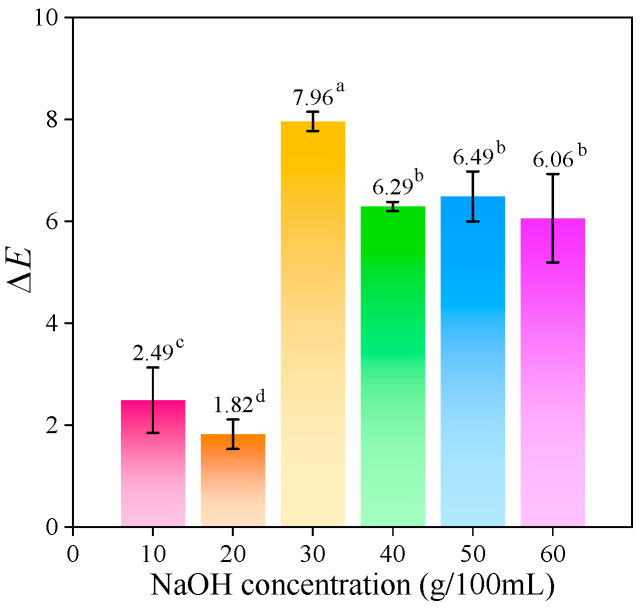
Study of the color measurement of cellulose and CMC_d_. Note: values indicated with the same letters are not significantly different at *p* ≤ 0.05.

The color measurement of CMC_d_ in Figure 4 demonstrates the color change in cellulose fibers and CMC_d_, impacted by chemical processes with DS via adding NaOH modification of cellulose fibers [1,14]. These color changes could be assessed using color reader analysis instruments. The color change observed in CMC_d_ was a result of the chemical reactions during the carboxymethylation process, which were influenced by the varying NaOH treatment concentrations (10–60% *w*/*v*).

The NaOH, used as an alkali for the DS in the carboxymethylation process, caused degradation of the cellulose structure, leading to color changes. The NaOH concentration effect significantly influenced the color intensity, with higher NaOH concentrations (30–60% *w*/*v*) producing the darker shades of CMC_d_. This dynamic color intensity with NaOH concentration reflected the processing of chemical processes on the fiber structure, as present in Table 1.

The color results revealed that cellulose fibers showed the highest L* at 93.49, indicating their brightness, with neutral a* and b* values. The brightness (L*) of CMC_d_ gradually decreased from 93.49 to 90.94 with increasing NaOH concentration, indicating that higher alkali levels led to a slight darkening of the cellulose fibers during their transformation into CMC_d_. By comparison, both a* (redness) and b* (yellowness) gradually increased with the rising NaOH concentrations from 0.64 to 1.12 and 6.49 to 14.12, respectively, caused by the DS effect in cellulose fibers. The Δ*E* values represented the total color difference improvement with higher NaOH concentrations, displaying the largest Δ*E* (7.96) in the CMC_d_-30 condition as a significant color change compared to cellulose fibers. Figure 4 depicts the Δ*E* values of CMC_d_-40, CMC_d_-50, and CMC_d_-60 samples, clearly showing the reduction in color-changing values, resulting in the higher NaOH concentration in CMC_d_ production [9]. These results suggested that the NaOH concentration plays a critical role in altering the chemical structure of fibers, expressing itself through their color [69].

### 2.2. CMC_d_/PVA Hydrogel Characterization

#### 2.2.1. Crosslinking Percentage of CMC*d*/PVA Hydrogel

**Figure 5:**
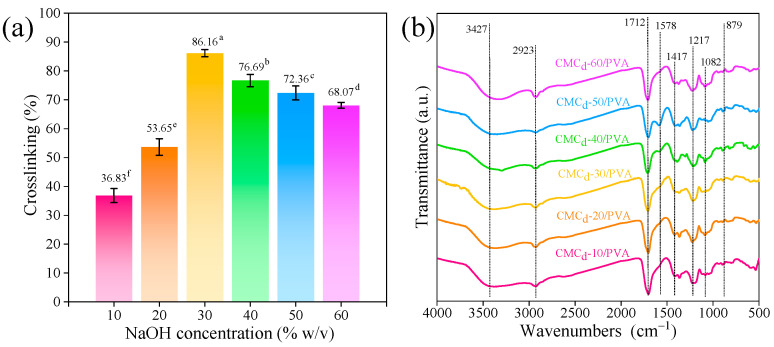
(**a**) Crosslinking percentage measurement and (**b**) FTIR results of CMC_d_/PVA hydrogel films with varied concentrations of NaOH for 10–60% *w*/*v*. Note: values indicated with the same letters are not significantly different at *p* ≤ 0.05.

Figure 5a shows the gel-fraction of CMC_d_/PVA hydrogel films prepared with varying NaOH concentrations. The crosslinking percentage gradually increased with NaOH concentrations from 36.83 to 86.16% for using 10 to 30% *w*/*v* of NaOH concentrations, then reduced to 68.07% upon a higher NaOH concentration usage (40–60% *w*/*v*) [70]. These results observed that the enhanced NaOH concentrations significantly improved the crosslinking interactions within the hydrogel matrix through various NaOH concentrations. The suitable NaOH concentration at 30% *w*/*v* could maximize the crosslinking percentages in CMC_d_-30/PVA at 86.16%, contributing to the stability of swelling properties mechanisms [25,71]. Additionally, the highest crosslink in CMC_d_-30/PVA hydrogel was affected by the maximum DS (0.94) from NaOH treatment, inducing more reactive sites with citric acid crosslinker. Carboxymethylation (higher DS) increases CMC_d_ solubility and chain expansion, improving citric acid diffusion and access to available hydroxyl groups. Although each substituted glucopyranose unit loses one original hydroxyl, the overall network at DS = 0.94 exhibits enhanced swelling and porosity, exposing a larger fraction of residual hydroxyls for esterification [54]. Carboxymethyl groups also introduce negative charges that promote electrostatic repulsion between chains, further opening the polymer matrix and facilitating uniform crosslinker distribution. These results enhanced the stable and interconnected polymer network strength of hydrogels, resulting in high crosslinking density and facilitating the usage of hydrogels in various applications. Corresponding to the highest crosslink in CMC_d_/PVA hydrogel films, they are continuously analyzed with FTIR and XPS spectroscopy, observing the molecular interactions spectra and the element binding energy information with an XPS high resolution of C 1s and O 1s in a hydrogel network structure.

#### 2.2.2. Chemical Functional Group of CMC_d_/PVA Hydrogels

The CMC_d_/PVA hydrogel films were examined using FTIR, illustrating the molecular interactions spectra of hydrogel films with various NaOH concentrations (10–60% *w*/*v*), as shown in Figure 5b. The functional groups of CMC_d_/PVA hydrogels were identified at 3427 and 2923 cm^−1^, ascribed to the presence of O–H and C–H stretching vibrational [25], respectively. CH_2_ in-plane vibrational, CH_2_ wagging, and asymmetric stretching C–O–C pyranose ring were identified in [25,52,53,54,55,56]. Then, the vibrational modes at 1082 and 879 cm^−1^ were classified as the molecule stretching of –C–O (C3–OH secondary alcohol) and –C–O stretch (C6–OH primary alcohol) [55], respectively.

In the FTIR spectrum of CMC_d_/PVA hydrogels (Figure 5b), the bands at 1578 and 1417 cm^−1^ correspond to the asymmetric and symmetric stretching vibrations of the carboxylate anion (–COO^−^), while the band at 1217 cm^−1^ is attributed to the asymmetric stretching of the C–O–C pyranose ring. The vibrations at 1712, 1578, 1417, and 1217 cm^−1^ are identified for carbonyl (C=O), CH_2_ in-plane vibrational, CH_2_ wagging, and asymmetric stretching C–O–C pyranose ring, respectively. However, a new peak at 1712 cm^−1^ also appears, attributable to ester carbonyl (C=O) stretching, which results from the esterification of CMC_d_ during crosslinking with citric acid.

The band at 1578 cm^−1^ arises from the asymmetric stretching vibration of –COO^−^ groups formed during the carboxymethylation of cellulose; its intensity tracks the DS. As NaOH concentration increases from 10 to 30% *w*/*v*, the cellulose fibers swell more effectively and yield a higher DS (up to 0.94), generating more –COO^−^ sites and thus amplifying the 1578 cm^−1^ signal. At concentrations above 30% *w*/*v*, however, excessive base induces partial chain scission and sodium carbonate/bicarbonate side-products, which consume or shield carboxylate moieties and disrupt the hydrogen-bond network, leading to a decline in peak intensity. This DS-dependent trend in carboxylate absorption has been documented in FTIR studies of CMC materials [50,51]. Enhanced intensity at 1217 and 1082 cm^−1^ (C–O–C stretching) further corroborates the establishment of ester linkages.

These results demonstrate that increasing the NaOH concentration significantly alters the chemical composition, crosslink density, and structural stability of CMC_d_/PVA hydrogel films [72,73,74,75,76].

The FTIR spectra of CMC_d_/PVA hydrogels exhibit clear markers of esterification and network formation. A new carbonyl stretching band appearing at ~1712 cm^−1^ confirms the formation of ester linkages between citric acid carboxyls and glucopyranose hydroxyls. Concurrently, there is a slight shift and broadening of the O–H stretching band (~3427 cm^−1^), consistent with enhanced hydrogen-bonding in the cross-linked network. A downshift of the C–O–C vibration from 1098 to 1082 cm^−1^ indicates the perturbation of glycosidic linkages due to ester bridge formation. These spectral changes, absent in uncross-linked CMC_d_/PVA, support successful cross-linking rather than mere component presence. Thus, the hydrogels synthesized from CMC_d_ with the high DS displayed stronger and more distinct absorption bands of these functional groups, indicating more extensive crosslinking and chemical modification aligned with the DS level.

#### 2.2.3. XPS Analysis of CMC_d_/PVA Hydrogel

**Figure 6:**
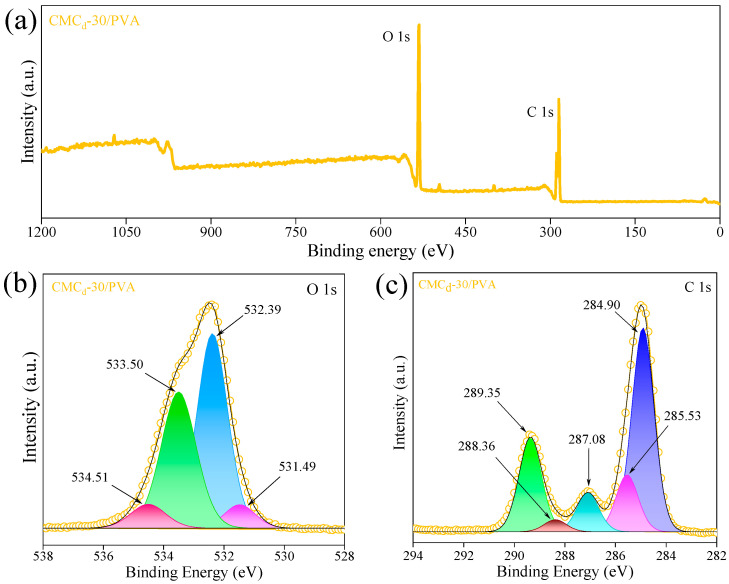
(**a**) XPS survey (**b**) O 1s and (**c**) C 1s spectra of CMC_d_-30/PVA hydrogel.

The CMC_d_-30/PVA hydrogel was used to investigate the surface signals using XPS spectroscopy, examining the incorporated C 1s and O 1s functional groups of hydrogels for this condition. The survey scan of binding energy at 1 to 1200 eV was performed to identify elemental composition (Figure 6a), followed by high-resolution scans for C 1s and O 1s core levels to analyze chemical bonding states. The high-resolution spectra of XPS for CMC_d_-30/PVA hydrogel were analyzed using deconvolution fitting through the Gaussian function, expressing the different oxidation states and functional groups in hydrogels. These results were related to the binding energy to confirm the material composition, chemical interactions, and evaluate crosslinking efficiency. The C 1s spectrum exhibited distinct peaks at 284.90, 285.53, 287.08, 288.36, and 289.35 eV (Figure 6b), corresponding to C–C/C–H [77], C–O/C–O–C, C–O of PVA [78], C=O from CA crosslinking [79,80], and O–C=O provided from carboxyl bonds [81], respectively. The O 1s spectrum in Figure 6c is displayed at 531.49, 532.39–533.50, and 534.51 eV, identifying as C=O [82,83], C–O–H [84], and C–O–C bonds [85], respectively [86].

The chemical shifts observed in the spectra indicate strong intermolecular interactions, verifying the structurally stable and chemically functionalized hydrogel network. The XPS spectra confirm successful crosslinking within the hydrogel network. The presence of ester (COO^–^) and carboxyl (COOH) peaks, along with their binding energy shifts, supports stable and hydrophilic polymeric network formation. Furthermore, the XPS peaks of CMC_d_-30/PVA hydrogel presented the effective crosslinking density, indicating the highest DS in CMC_d_-30 and enhancing the chemical bonding and strength network of the hydrogel structure. These findings indicated effective crosslinking and functionalization, making hydrogel suitable for applications through high water retention, structural stability, and bioactivity.

#### 2.2.4. Thermal Properties Comparison of CMC_d_ and CMC_d_/PVA Hydrogels

**Figure 7:**
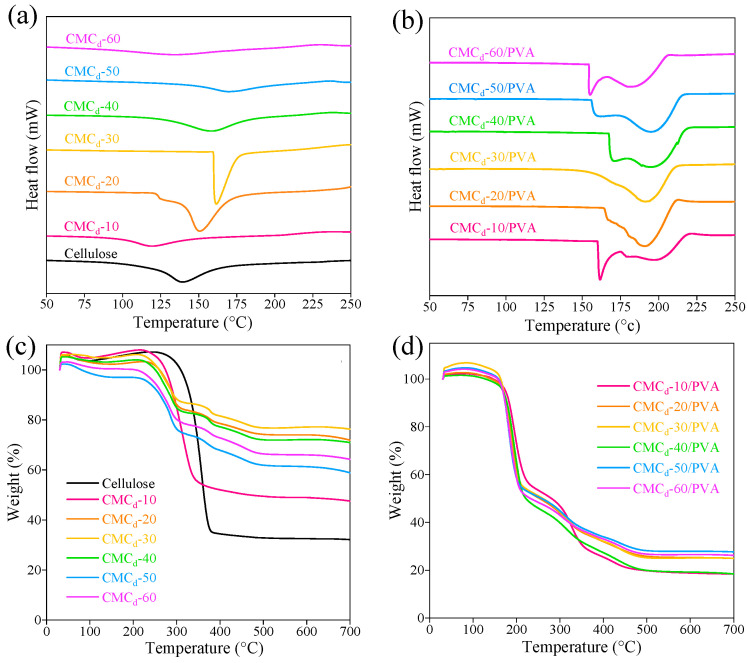
DSC results of (**a**) CMC_d_, (**b**) CMC_d_/PVA hydrogel films, TGA results for thermal degradation behavior of (**c**) CMC_d_, and (**d**) CMC_d_/PVA hydrogel films with various conditions.

The DSC result in Figure 7a illustrates the thermal behavior of durian rind cellulose and CMC_d_, indicating the progressive thermal transition from durian rind cellulose at 139 °C, verified as the thermal degradation temperatures. The CMC_d_-10 and CMC_d_-20 demonstrated the melting point temperature at 117 °C and 150 °C, respectively. CMC_d_-30 exhibited an endothermic melting/crystalline transition at 161 °C, resulting in high thermal energy consumption, attributed to the existence of a crystalline uniformity structure formation via thermal stability enhancement, as well as the XRD results in Figure 1b.

The decreased thermal stability of CMC_d_-40 through CMC_d_-60 arises from over-carboxymethylation, which disrupts intermolecular hydrogen bonding and crystalline domain integrity, leading to diminished melting and degradation transitions. All CMC_d_ samples were washed with water until near-neutral pH prior to thermal analysis to minimize the presence of residual sodium salts. However, as shown in the XRD results, traces of NaHCO_3_ were still detected, likely due to secondary reactions between residual NaOH and atmospheric CO_2_ during drying and storage.

Figure 7b displays the DSC results of CMC_d_/PVA hydrogel films, comparing the thermal behavior of hydrogel films in each condition. The hydrogel films of CMC_d_-10/PVA and CMC_d_-20/PVA were observed to have two distinct denaturation temperatures in the range of 161 to 197 °C, caused by the two different crystalline phases forming in the hydrogel structure conditions. These hydrogel conditions revealed the incomplete polymer interaction due to their insufficient crosslinking density for the hydrogel film formation as a homogeneous network [14]. The DSC result of CMC_d_-30/PVA hydrogel exposed the transition of melting point temperature at 192 °C, implying the existence of a homogeneous phase and having a high crosslinking density in this hydrogel condition. The hydrogel films of CMC_d_-40/PVA to CMC_d_-60/PVA demonstrated the two distinct melting temperatures around 154 to 195 °C, indicating the separated crystalline phase of these hydrogels and disrupting the homogenous phase formation of the hydrogel [58]. The DSC thermogram of CMC_d_-30/PVA displays a single dominant endothermic transition centered at 192 °C with moderate breadth, indicative of a structurally heterogeneous yet well-crosslinked semi-crystalline network. This thermal behavior reflects high crosslinking density, which is consistent with the optimal DS that promotes enhanced structural integrity and thermal stability.

The TGA results in Figure 7c exhibit the behavior of cellulose and CMC_d_ treated at temperatures ranging from 30 to 700 °C under different CMC_d_ conditions. The TGA results indicated that cellulose was converted into CMC_d_ through the carboxymethylation process, facilitated by NaOH, which provides an alkaline environment for the reaction. This enhances the thermal stability compared to cellulose, inducing this CMC_d_ to more significantly retain weight at higher temperatures. With the low temperature (less than 200 °C), CMC_d_-10 showed the outstanding thermal stability compared to other samples. When the temperature increased from 200 to 330 °C, cellulose and CMC_d_-10 displayed the highest thermal resistance compared to other samples at the same temperature. The highest stability of CMC_d_-10 was attributed to the larger cellulosic content in the component, which enhanced the resistance to thermal decomposition. At high temperatures (330–700 °C), CMC_d_-30 gradually reduced weight loss with stable thermal resistance compared to cellulose and other CMC_d_ conditions. The stability of CMC_d_-30 was observed with the growth of crystalline and inter-bonding forces, resulting in better thermal resistance and providing the highest thermal stability with their higher crystallinity improved [87], as shown in the TGA result.

The hydrogel films in Figure 7d illustrate the correlation of TGA results with CMC_d_/PVA hydrogel films composed of CMC_d_ as a component, revealing distinct thermal degradation patterns. By the transition from 150 to 200 °C, all hydrogel films rapidly diminished in weight from 97 to 50% of this scope temperature, then the weight gradually reduced from 200 to 500 °C and stabilized at temperatures higher than 500 °C.

The weight loss observed between 150 °C and 200 °C (~50%) corresponded to the evaporation of residual moisture in the hydrogel films. This residual moisture likely remained within the hydrogel matrix, despite the rinsing procedure, and contributed to the initial weight loss during thermal analysis. While at 200 to 500 °C, it was obviously pronounced from the hydrogel film degradation depending on the various crosslinking densities of each hydrogel condition. Between 200 and 500 °C, CMC_d_-10 and CMC_d_-40 hydrogel films exhibited more rapid weight loss, indicating lower crosslink density and increased polymer decomposition.

The TGA analysis also revealed that CMC_d_-30 exhibited the highest onset degradation temperature (T_onset_ = 161 °C) and the largest peak in the derivative thermogravimetry curve, indicating optimal thermal resistance due to balanced carboxymethyl substitution and preserved crystallinity. However, its residual weight at 700 °C is comparable to that of CMC_d_-20 and CMC_d_-40. By the thermal degradation of hydrogel films in this work, CMC_d_/PVA hydrogel films were enhanced in thermal resistance and stability using the addition of CMC_d_ as a component, contributing to the crosslinking structure development of hydrogel formation. The maximum degradation temperatures (T_max_), extracted from the derivative TGA curves, were reported as follows: CMC_d_-10/PVA (314 °C), CMC_d_-20/PVA (328 °C), CMC_d_-30/PVA (342 °C), CMC_d_-40/PVA (336 °C), CMC_d_-50/PVA (330 °C), and CMC_d_-60/PVA (324 °C). These values quantitatively confirm that the CMC_d_-30/PVA film exhibits the highest thermal stability, reflecting its optimal crosslink density and enhanced crystallinity, relating to the network structure of ICTAC kinetics Committee recommendations for thermal analysis [88].

This enhanced stability is attributed to its high DS, which facilitates stronger intermolecular bonding and improved crystalline uniformity, thereby increasing resistance to thermal degradation.

### 2.3. MB Releasing Ability and Kinetic

#### 2.3.1. Swelling Ratio of CMC_d_/PVA Hydrogel Films

**Figure 8:**
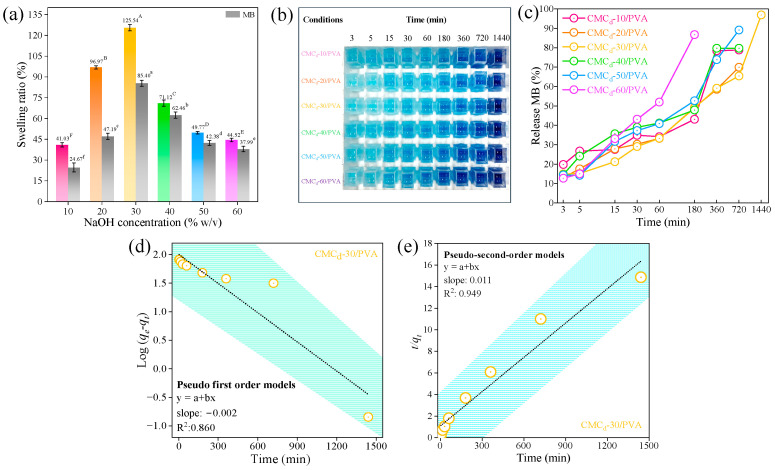
(**a**) Swelling ratio results from treated and untreated MB of CMC_d_/PVA hydrogels, (**b**) MB release testing, (**c**) MB releasing results using kinetic model of (**d**) pseudo-first order, and (**e**) second-order models. Note: values indicated with the same letters are not significantly different at *p* ≤ 0.05.

The swelling testing of hydrogel films was conducted using CMC_d_/PVA hydrogel films, studying the mechanism for MB absorption. The swelling results of each CMC_d_/PVA hydrogel film in Figure 8a were correlated with crosslinking density in Figure 5a, revealing the increased swelling ratio like the increased crosslinking density on each hydrogel film’s condition. The swelling ratio results were enhanced in the hydrogel films using CMC_d_-10, CMC_d_-20, and CMC_d_-30 as components and subsequently reduced upon adding CMC_d_-40 and CMC_d_-60, corresponding to the crosslinking density factor for maximum water uptake [89]. By the swelling comparison on MB absorption, the overall hydrogel films with MB treatment had reduced swelling ratio properties, influenced by their hydrogel swelling capacity of each hydrogel condition based on the different crosslinker density [90,91]. CMC_d_-30/PVA displayed the outstanding swelling ratio at 125.54%, supporting the enhancement of its MB absorption ability with an impact of highly crosslinking and leading to the domination change in swelling ratio after MB treatment [81,92].

The behavior of hydrogels is correlated with their high DS, supporting the availability of a hydrophilic property allowing greater water absorption and expansion. CMC_d_-30/PVA displayed the highest swelling ratio (125.54%), which arises from a balance between increased network crosslinking and exceptionally high hydrophilicity due to carboxymethyl substitution. At DS ≈ 0.67, the abundance of –COO^−^ groups generates strong osmotic driving forces that overcome the network tightening effect, promoting maximal water uptake. In samples with higher crosslink densities (CMC_d_-30), the swelling ratio was higher, while in samples with lower crosslink densities (CMC_d_-40 and CMC_d_-60), the swelling ratio decreased despite their hydrophilic nature, due to the reduced gel fraction and mesh size [93,94].

#### 2.3.2. Methylene Blue Releasing

The MB release results illustrated the release behavior of MB in CMC_d_/PVA hydrogel films under various conditions. Figure 8b presents the cumulative release of MB from CMC_d_/PVA hydrogel films into PBS (pH 7.4) The cumulative release of MB was observed at 180 min, showing the released MB of 40–50% *w*/*v* in each hydrogel condition, while CMC_d_-60/PVA revealed over 80% of released MB before the process was stopped. The highest released MB efficiency was highly observed at 360–720 min, indicating 60–90% of released MB, and immediately stopped after 720 min. The CMC_d_-30/PVA hydrogel condition was achieved with the highly stable release of MB up to 1440 min with 100% of released MB efficiency (Figure 8c).

To study the mechanism of MB releasing, the pseudo-first-order kinetics model [95] was used to study the MB releasing kinetics in hydrogels under each condition, as shown in Equation (1).

(1) $$\log\left(q_{e}-q_{t}\right)=logq_{e}-\frac{K_{1}t}{2.303}$$

where *q_t_* (mg/g) and *q_e_* (mg/g) are the amount of dye adsorbed at time “*t*” and equilibrium. *K*_1_ is the pseudo-first-order rate constant in 1/min. The graph of *log*(*q_e_* − *q_t_*) versus t gives a straight line with *K*_1_ as the slope and *q_e_* as the intercept [96].

CMC_d_-30/PVA was chosen to determine the kinetic correlation of pseudo-first order with experimental data, as illustrated in Figure 8c. The linear correlation results of *log*(*q_e_* − *q_t_*) and time showed R^2^ at 0.860, which is not a good fit with the experimental data points. The linear correlation of this model was not enough to explain the releasing MB behavior from the CMC_d_-30/PVA hydrogel. The MB release of CMC_d_-30/PVA hydrogel was extended to 1440 min, sustained with a drug-releasing profile. This hydrogel condition is attributed to the highly dense crosslinking structure formed by CMC_d_-30 with a high DS in the component, resulting in restricted rapid diffusion and supporting gradual drug liberation. The pseudo-second-order kinetics model was subsequently studied for the releasing MB of CMC_d_-30/PVA hydrogel, as expressed in Equation (2) [97,98].

(2) $$\frac{t}{q_{e}}=\frac{1}{K_{\begin{array}{c} 2\\ \; \end{array}}q_{e}^{2}}+\frac{1}{q_{e}}$$

where *K*_2_ is pseudo-second-order rate constant (g/mg min) and *q_e_* is the equilibrium adsorption capacity. The *q_e_* and *K*_2_ are obtained from the slope and intercept of the plot *t*/*q_t_* versus *t* [96].

Figure 8e provides a good fit to the experimental data with the linear correlation of higher R^2^ at 0.949. The strong linearity was observed in the plot of *t*/*q_t_* versus time, indicating that this kinetic model could be anticipated. The pseudo-second-order model was effectively described as releasing MB kinetics, contributing to the promising properties for the drug delivery applications, as compared to other studies in Table 2. These MB releasing modes were modulated through the crosslinking density of hydrogels. The release kinetics of CMC_d_-30/PVA hydrogel could be applied to control and accelerate the release profile depending on the requirement in the application. The optimization of NaOH concentrations to CMC_d_-30/PVA hydrogel structure formation was targeted release capabilities, providing a versatile approach to tailor drug delivery, wound care, and other controlled-release applications.

## 3. Conclusions

In summary, this study has shown that carboxymethyl cellulose derived from durian rind at 30% NaOH (CMC_d_-30) achieves a degree of substitution of 0.94 and, when blended with PVA and crosslinked by citric acid, forms a hydrogel with outstanding crosslinking efficiency of 86.16%. This optimized CMC_d_-30/PVA hydrogel exhibits a high swelling ratio (125.54%), a single sharp melting transition at 192 °C, indicative of enhanced thermal stability, uniform porous morphology, and a sustained methylene blue release profile extending to 1440 min under pseudo-second-order kinetics. Together, these performance attributes highlight a value-added route for converting agro-waste into a robust, biocompatible hydrogel platform with clear promise for wound-dressing and controlled drug-delivery applications.

## 4. Materials and Methods

### 4.1. Materials

Durian rinds were collected from the Mon Thong breed in Uttaradit, Thailand. All chemical reagents were purchased from commercial suppliers; isopropanol (99.5%) and citric acid (CA) (99.5%) were provided by Loba Chemie Co., Ltd., Mumbai, India. NaOH from RCI Labscan co. Ltd., Bangkok, Thailand, monochloroacetic acid from Sigma Aldrich, St. Louis, MO, USA. Glacial acetic acid was provided from Qrec, Auckland, New Zealand. PVA (Mw ≈ 100,000 g/mol, degree of hydrolysis ~88–89%) was purchased from Chem Supply Co., Ltd., Gillman, Australia. MB from KemAus™, Cherrybrook, NSW, Australia.

### 4.2. Durian Rind Cellulose and CMC_d_ Preparation

Cellulose was extracted from durian peel by an alkaline treatment followed by the double bleaching process. Durian peel (100 g) was boiled in NaOH solution with 18% *w*/*v* concentration in water (2000 mL) at 85 °C for 5 h, then continuously stirred at 1000 rpm to remove hemicellulose and lignin components. The pulp was obtained by filtering and washing to neutral pH (pH ~ 7), then dried at 80 °C for 12 h. The treated pulp was bleached by an acetate buffer solution (pH ~ 4), prepared by mixing a solution of NaOH, acetic acid, and sodium chlorite for 5.4% *w*/*v*, 15% *v*/*v*, and 3.4% *w*/*v*, respectively [104]. The pulp was treated with an acetate buffer solution at 85 °C for 3 h using stirring at 1200 rpm repeated twice. The bleached cellulose was filtered and washed to neutral, then dried at 105 °C for 3 h, and stored in a desiccator to prevent moisture absorption [105].

CMC_d_ were synthesized by dispersing cellulose powder (15 g) in 50 mL of isopropyl alcohol containing sodium hydroxide (10–60% *w*/*v*). Subsequently, monochloroacetic acid (18 g) was added, and the mixture was stirred at 55 °C for 3 h to complete the carboxymethylation reaction. The sample solution formed a suspension, and the liquid and solid phases were then separated by filtration. The filtered sample was washed with methanol 5 times. The residue solid phase of the sample was filtered and dried overnight at 55 °C using a hot air oven, obtaining a yield product of CMC_d_ in powder from this process [1,106]. The CMC_d_ yields were calculated using Equation (3), comparing the initial amount of cellulose and CMC_d_. All experiments, including the synthesis and yield calculations, were performed in triplicate to ensure the accuracy and reliability of the results.

(3) $$CMC\;yield\left(\%\right)=\frac{Weight\;of\;CMC\;(g)}{Weight\;of\;Cellulose\;(g)}\times 100$$

#### 4.2.1. Degree of Substitution

The degree of substitution (DS) was determined following the procedure outlined in the USP XXII method specified for croscarmellose sodium. This involved a series of steps, including titration and determination of residue on ignition, which have been extensively elucidated in other sources [1]. The DS of the CMC_d_ in this study was determined by back-titration of free carboxylic groups (M, net milliequivalents of base per gram of CMC_d_) combined with residue-on-ignition analysis (C, percentage *w*/*w*). The total DS was separated into acid-form (A) and sodium-form (S) contributions, calculated by Equation (4):

(4) DS=A+S

(5) $$A=\frac{1150M}{\left(7120-412M-80C\right)}$$

(6) $$S=\frac{\left(162+58A\right)C}{\left(7120-80C\right)}$$

where 7120 mg corresponds to the theoretical mass of 100 anhydroglucose units (162 g mol^−1^ × 1000 mg/g × 100 g/162 g), 412 mg is the equivalent mass per mg of the acid carboxymethyl substituent (–CH_2_–COOH; 58 g mol^−1^, plus a minor correction for stoichiometry), and 80 mg is the equivalent mass per mg of the sodium carboxymethyl substituent (–CH_2_–COONa; approx. 80 g mol^−1^). Thus, Equation (5) converts the titration result (M) into the number of acid substituents per anhydroglucose unit, accounting for mass changes due to substitution and ignition residue. Equation (4) then apportions the remaining substitution into sodium form based on the ignition loss (C). A and S are the degree of substitution for acid carboxymethyl and sodium carboxymethyl, estimated using Equations (5) and (6), respectively. M is required to neutralize all of the acidic carboxymethyl groups present in exactly one gram of dried CMC_d_, as determined by back-titration. Expressing M per gram of sample standardizes the titration result for use in Equations (5) and (6) to calculate the degree of substitution. C is the residue percentage on ignition of CMC_d_ as determined in residue on ignition testing.

#### 4.2.2. Crosslinking Percentage

The hydrogel films (1 × 1 cm) were immersed in water and stirred for 12 h to dissolve any unreacted hydrogels. The residue hydrogels were filtered and washed with water and acetone, respectively. Hydrogels were dried overnight at 40 °C, then the percentage crosslinking was estimated using Equation (7) [25].

(7) $$Crosslinking\left(\%\right)=\frac{W_{1}}{W_{2}}\times 100$$

where *W*_1_ and *W*_2_ are the weights of the dried films before and after dissolution, respectively. The crosslinking measurements were repeatedly analyzed and averaged 5 times [71].

### 4.3. Characterization of CMC

#### 4.3.1. Morphology Investigation CMC_d_

Assessment of the surface morphology was conducted using a scanning electron microscope (SEM) at 10 kV (JSM-IT300LV model by JEOL Co., Ltd., Tokyo, Japan), providing detailed insights into the structure and surface characteristics of the CMC_d_ powders [107].

#### 4.3.2. Structural Analysis of CMC_d_

Using X-ray diffraction (XRD), the crystallographic structure, phase composition, and crystallinity of the synthesized CMC_d_ material [108] were characterized. The XRD measurements were carried out using a spectrometer (Rigaku MiniFlex, Tokyo, Japan) to record the diffraction signal over the 2θ range from 10 to 60 degrees at a scan speed of 2°/min [109].

#### 4.3.3. Functional Group of CMC_d_

Fourier transform infrared spectroscopy (FTIR) was conducted to analyze the functional groups of the CMC_d_ samples, using a spectrophotometer (FT/IR-4700, JASCO International Co., Ltd., Pfungstadt, Germany). Cellulose and CMC_d_ samples were mixed with KBr and pressed into pellets. The transmission results of FTIR were measured in wavelengths of 4000 to 400 cm^–1^ [110].

#### 4.3.4. X-Ray Photoelectron Spectroscopy

X-ray photoelectron spectroscopy (XPS) was used to collect the surface signal from the monochromatic Al K excitation with 20 eV (scan rate: 100 meV), measured by AXIS Ultra DLD, Kratos Analytical Co. Ltd., Manchester, England. CMC_d_/PVA hydrogels were used to analyze the chemical bonding and elemental composition on the surface of the sample.

#### 4.3.5. Viscosity and Water Solubility of CMC_d_ Samples

CMC_d_ samples (1 g) were dissolved in water solutions (25 mL) and then continuously stirred at 500 rpm for 1 h at room temperature [1,5,106]. The viscosity was measured at 200 rpm for 10 s under 25 °C using a Brookfield viscometer (LVDV-2, Newport Scientific, Middleboro, MA, USA). In the water solubility measurement, the CMC_d_ samples were dried at 60 °C for 24 h to eliminate any residual moisture. The dried samples were transferred to Erlenmeyer flasks (250 mL) containing 50 mL of distilled water. The flask was stirred at 25 rpm for 24 h and sonicated to ensure uniform dissolution. The supernatant samples were filtrated to separate the solid residue, then collected and dried with a hot-air oven at 80 °C for 24 h to remove water content. The water solubility percentage was calculated using Equation (8) by comparing the weight of the dissolved material (W*f*) to the weight of the original sample (W*i*) [111,112].

(8) $$Solubility\;(\%)=\left|\frac{W_{i}-W_{f}}{W_{i}}\right|\times 100$$

#### 4.3.6. Thermal Properties

Thermal analysis of CMC_d_ and CMC_d_/PVA hydrogel samples was conducted to evaluate their thermal behavior and stability using differential scanning calorimetry (DSC; Mettler Toledo DSC1, Columbus, Ohio, USA. The DSC analysis of samples (5 mg) placed in a sample pan measures the calorific value depending on temperature changes and examines the material structure transitions compared to a standard reference substance. The thermogravimetric analysis (TGA) of the samples was carried out at 30–350 °C with a 10 °C/min heating rate under a nitrogen atmosphere using the Mettler Toledo Stare System TGA/DSC3+ Module-Auto Sample Robot, Columbus, Ohio, USA [113]. The nitrogen gas was used to control the oxidation process, providing accurate thermal characterization. TGA samples were heated at 25–700 °C, assessing chemical, physical, and structural transformation of material as a function of temperature [87]. These thermal analysis techniques monitored the material weight loss under heating and revealed the energy of phase transitions under the calorimetric effect. Both DSC and TGA provided comprehensive insights into the thermal stability, energy absorption, and decomposition patterns of the sample films [114].

#### 4.3.7. Swelling Ratio Analysis

The swelling behavior of the hydrogel films was investigated in deionized (DI) water at 37 °C. The hydrogel films (0.2 g) were submerged in DI water (20 mL) for 30 min. The swollen film samples were extracted from the DI water to remove excess water and weighed on an analytical balance. The swelling ratio of the hydrogel films was calculated using Equation (9) as follows.

(9) $$Swelling\;Ratio=\frac{W_{s}-W_{d}}{W_{d}}\times 100$$

where *W_d_* and *W_s_* represent the weights of dry and wet hydrogel, respectively [81].

#### 4.3.8. Color Measurement

The color properties of cellulose and CMC_d_ samples were analyzed using a Color Quest XE Spectrocolorimeter (Hunter Lab Colorflex EZ 45-0 (LAV), Shen Zhen Wave Optoelectronics Technology Co., Ltd., Xiamen, China). The measurements were conducted in the CIELAB color space, described by three parameters (L*, a*, and b*). L* indicates lightness from black (0) to white (100), a* denotes the spectrum between greenness (−) and redness (+), and b* represents the spectrum from blue (−) to yellow (+). The overall color difference (∆E) was determined by the L*, a*, and b* values of samples using the formula specified in Equation (10) [9].

(10) $$∆E=\sqrt{{\left(∆L^{*}\right)}^{2}{+(∆a^{*})}^{2}{+(∆b^{*})}^{2}}$$

### 4.4. Preparation of CMC_d_/PVA Hydrogel Films

PVA was dissolved in water at 50 °C under stirring for 20 min. CMC_d_ was added to the PVA solution at room temperature. Hydrogel films were prepared using PVA, CA, and CMC_d_ at varying NaOH concentrations. The CMC_d_/PVA hydrogel films were fabricated by combining CMC_d_ (2% *w*/*v*), prepared under various NaOH concentrations (10–60% *w*/*v*), with PVA (2% *w*/*v*) and CA (6% *w*/*v*) as the crosslinking agent [53]. The sample solution was stirred to achieve a homogeneous solution overnight to remove air bubbles. The homogeneous solution was poured into Petri dishes (9 cm^2^) and dried in a hot-air oven at 50 °C for 24 h. The dried CMC_d_/PVA films were cured at 80 °C for 5 min to enhance the crosslinking between the polymer chains, improving the structural integrity of the hydrogel films. The hydrogel films were neutralized by washing with distilled water, then rinsed with isopropyl alcohol to remove entrapped water from the matrix films [25].

### 4.5. In Vitro Release Study of Model Drugs

The released MB amount was quantified using UV-visible spectrophotometer (UV-Vis; Jasco V-730, JASCO International Co., Ltd., Hachioji, Japan,) at 644 nm absorption wavelength. The cumulative drug release was calculated using Equations (9)–(11) [115,116].

Square-shaped CMC_d_/PVA hydrogel films (1 × 1 cm, dry mass ≈ 10 mg) were first loaded with MB by immersion in 10 mL of 0.1 mg/mL MB solution for 24 h at room temperature. Release studies were conducted in 20 mL of phosphate-buffered saline (PBS, pH 7.4) under semi-static conditions at 25 °C. At predetermined time points (3, 5, 15, 30, 60, 180, 360, 720, 1440 min), 1 mL aliquots of release medium were withdrawn and immediately replaced with 1 mL fresh PBS to maintain sink conditions [117].

The released MB amount was quantified using UV-visible spectroscopy (Jasco V-730, JASCO International Co., Ltd., Japan, UV-Vis) at 644 nm absorption wavelength. The cumulative drug release was calculated using Equations (11)–(13) [115,116]. The MB concentration in each aliquot (*C_MB_*, µg/mL) was quantified by UV–Vis spectrophotometry at 644 nm, the long-wavelength absorption maximum of MB in aqueous media. A calibration (standard) curve was prepared by measuring the absorbance of MB standard solutions (0–50 µg/mL) at 644 nm, and its slope (*k*, absorbance per µg/mL) was used to convert absorbance to the concentration in Equation (11).

(11) $$C_{MB}=A_{644}/k\;$$

where *A*_644_ is the measured absorbance at 644 nm. The amount of MB released in each sample (Drug content) was calculated by multiplying *C_MB_* by the sample volume (*V_sample_*: 1 mL). No further dilution was performed before measurement, resulting in a dilution factor (DF) of unity. Cumulative percent release (% Release) was then calculated by summing the released amounts and normalizing to the theoretical MB content loaded per film (based on initial loading conditions and film mass) as follows [98]:

(12) $$Drug\;content\;(µg)=C_{MB}\times V_{sample}$$

(13) $$\%\;Release=\frac{Drug\;content}{Theoretical\;content\;loaded}\;\times 100$$

This calculation approach follows standard practice for drug release from hydrogels, as described by Cesco et al. for MB-loaded polysaccharide networks.

### 4.6. Statistical Analysis

The results were analyzed using one-way ANOVA conducted in SPSS 17.1 software. Statistically significant differences were determined at a 95% confidence interval (*p* < 0.05) using the Duncan multiple range test among the five samples [118].

## Figures and Tables

**Table 1:** Color tracking results of natural cellulose fibers and CMC_d_.

Sample	L*	a*	b*	ΔE
Cellulose	93.49 ± 0.14	0.64 ± 0.01	6.49 ± 0.13	-
CMC_d_-10	92.14 ± 0.78	0.94 ± 0.07	8.56 ± 0.18	2.49 ^c^ ± 0.64
CMC_d_-20	92.37 ± 0.43	0.77 ± 0.02	7.92 ± 0.16	1.82 ^d^ ± 0.29
CMC_d_-30	91.22 ± 0.32	0.69 ± 0.04	14.12 ± 0.19	7.96 ^a^ ± 0.19
CMC_d_-40	91.95 ± 0.06	1.12 ± 0.04	12.57 ± 0.12	6.29 ^b^ ± 0.09
CMC_d_-50	90.94 ± 0.62	0.97 ± 0.04	12.45 ± 0.02	6.49 ^b^ ± 0.49
CMC_d_-60	92.29 ± 0.85	0.86 ± 0.06	12.39 ± 0.51	6.06 ^b^ ± 0.87

Note: values indicated with the same letters are not significantly different at *p* ≤ 0.05.

**Table 2:** Comparison of the MB releasing ability in hydrogels.

Samples	Structure	Releasing Time (min)	Reference
Carboxymethyl sago pulp (CMSP)	CMSP hydrogel nanofibers	2880	[99]
Carboxymethyl cellulose and chitosan	Complex beads	360	[100]
Polyvinyl alcohol/agarose/maltodextrin composite and durian peel (DP)	Composite membrane	300	[101]
Carboxymethyl cellulose-g-poly (acrylamide-co-methacrylic acid)/ Cloisite 30B	Nanocomposite hydrogel	120	[102]
Carboxymethyl cellulose nanocrystal	Hydrogel	360	[103]
Carboxymethyl cellulose/Poly(vinyl) Alcohol	Hydrogel films	1440	Our work

## Data Availability

The original contributions presented in this study are included in the article. Further inquiries can be directed to the corresponding author(s).
